# Understanding the Acceptability of Subdermal Implants as a Possible New HIV Prevention Method: Multi-Stage Mixed Methods Study

**DOI:** 10.2196/16904

**Published:** 2020-07-27

**Authors:** Christine Tagliaferri Rael, Cody Lentz, Alex Carballo-Diéguez, Rebecca Giguere, Curtis Dolezal, Daniel Feller, Richard T D'Aquila, Thomas J Hope

**Affiliations:** 1 HIV Center for Clinical and Behavioral Studies New York State Psychiatric Institute Columbia University New York, NY United States; 2 Department of Biomedical Informatics Columbia University New York, NY United States; 3 Division of Infectious Diseases Northwestern University Feinberg School of Medicine Chicago, IL United States; 4 Feinberg School of Medicine Northwestern University Chicago, IL United States; 5 McCormick School of Engineering and Applied Sciences Northwestern University Chicago, IL United States

**Keywords:** PrEP implant, YouTube, acceptability, long-acting PrEP, systemic PrEP, human-centered design, HIV prevention, removable implant, long-acting HIV prevention, gay and bisexual men

## Abstract

**Background:**

A long-acting implant for HIV pre-exposure prophylaxis (PrEP) is in development in the Sustained Long-Action Prevention Against HIV (SLAP-HIV) trial. This could provide an alternative to oral PrEP.

**Objective:**

Our mixed methods study aimed to understand (1) users’ experiences with a similar subdermal implant for contraception and (2) factors influencing the likelihood that gay and bisexual men (GBM) would use a proposed PrEP implant.

**Methods:**

Work was completed in 4 stages. In stage 1, we conducted a scientific literature review on existing subdermal implants, focusing on users’ experiences with implant devices. In stage 2, we reviewed videos on YouTube, focusing on the experiences of current or former contraceptive implant users (as these implants are similar to those in development in SLAP-HIV). In stage 3, individuals who indicated use of a subdermal implant for contraception in the last 5 years were recruited via a web-based questionnaire. Eligible participants (n=12 individuals who liked implants a lot and n=12 individuals who disliked implants a lot) completed in-depth phone interviews (IDIs) about their experiences. In stage 4, results from IDIs were used to develop a web-based survey for HIV-negative GBM to rate their likelihood of using a PrEP implant on a scale (1=very unlikely and 5=very likely) based on likely device characteristics and implant concerns identified in the IDIs.

**Results:**

In the scientific literature review (stage 1), concerns about contraceptive implants that could apply to the PrEP implants in development included potential side effects (eg, headache), anticipated high cost of the device, misconceptions about PrEP implants (eg, specific contraindications), and difficulty accessing PrEP implants. In the stage 2 YouTube review, individuals who had used contraceptive implants reported mild side effects related to their device. In stage 3, implant users reported that devices were comfortable, unintrusive, and presented only minor discomfort (eg, bruising) before or after insertion and removal. They mainly reported removing or disliking the device due to contraceptive-related side effects (eg, prolonged menstruation). Participants in the stage 4 quantitative survey (N=304) were mainly gay (204/238, 85.7%), white (125/238, 52.5%), cisgender men (231/238, 97.1%), and 42.0% (73/174) of them were on oral PrEP. Not having to take a daily pill increased the likelihood of using PrEP implants (mean 4.13). Requiring >1 device to achieve 1 year of protection (mean range 1.79-2.94) mildly discouraged PrEP implant use. Participants did not mind moderate bruising, a small scar, tenderness, or bleeding after insertion or removal, and an implant with a size slightly larger than a matchstick (mean ratings 3.18-3.69).

**Conclusions:**

PrEP implants are promising among GBM. Implant features and insertion or removal-related concerns do not seem to discourage potential users. To ensure acceptability, PrEP implants should require the fewest possible implants for the greatest protection duration.

## Introduction

Subdermal implants represent a promising, long-acting biomedical HIV prevention innovation in the development pipeline. A total of two trials are currently investigating subdermal implants for HIV pre-exposure prophylaxis (PrEP): (1) the Sustained Long-Acting Prevention against HIV (SLAP-HIV) trial (preclinical phases) [1,2] and (2) Centre for the AIDS Programme of Research in South Africa-018 (phase I or II) [3]. Little is known about whether such HIV prevention devices would be acceptable or feasible for end users [4]. This is a concern given that uptake of existing PrEP products (eg, oral PrEP) is suboptimal in some key populations who are disproportionately affected by the HIV epidemic. For example, in a 2017 cohort study of 995 gay and bisexual men (GBM) across the United States, only 4% of participants reported currently using oral PrEP [5]. The reasons for this limited uptake are continually emerging [6-9]. Involving potential consumers in the initial stages of product development could help mitigate later issues with method use. Specifically, early consumer involvement could help researchers understand end users’ anticipated challenges with uptake and adherence, allowing them to address and potentially limit these problems in the design of clinical tools [10].

SLAP-HIV is an interdisciplinary research project that aims to identify, develop, and test a long-acting PrEP implant. A proposed version of this product could be inserted under the skin of a user’s upper arm. This device is conceptualized to slowly dispense a single HIV prevention medication (such as tenofovir alafenamide or cabotegravir) over a period (eg, 2-12 months). Similar devices are currently used by cisgender women (ie, individuals who were assigned female at birth and identify as women) to dispense contraceptive medication (eg, Nexplanon) [11]. Thus, it is possible to learn from the experiences of current and past users of an implant similar to the one in development to understand their likes and dislikes about this mode of drug delivery.

This study was undertaken as part of SLAP-HIV. We aimed to identify barriers and facilitators anticipated by GBM who might use PrEP implants in the future. Although the end goal of this work focused on the opinions of GBM, we focused on cisgender women during the initial stages, as the contraceptive implant is the product most similar to the one in development by SLAP-HIV.

## Methods

This work was completed in 4 stages, which are summarized below.

### Stage 1: Scientific Literature Review

We conducted a review of the scientific literature on publicly available (eg, Google Scholar and PubMed) and university-specific (eg, Columbia University libraries) databases to identify barriers and facilitators of contraceptive implant use among cisgender women. We focused our review on contraceptive implants, as this device is most similar to the one in development by SLAP-HIV. Furthermore, there is extremely limited data on cisgender men’s experiences with implants to deliver gender-specific medications (eg, the Testopel testosterone implant); these implants also operate differently than those under study in SLAP-HIV (eg, Testopel is a nonremoveable biodegradable pellet implant) [12]. We limited our search to studies published in the last 10 years (January 1998 to July 2017). Search terms included “contraceptive implant barriers,” “contraceptive implant removal,” “contraceptive implant facilitators,” “contraceptive implant experiences,” and “contraceptive implant continuation.” We limited the search to studies published about US populations and manuscripts on arm-implant contraceptives (eg, studies focused only on intrauterine devices [IUDs] were excluded).

The primary author (CR) and a research assistant (CL) abstracted relevant studies and independently reviewed their full content to determine their applicability to the topic under study (eg, device-related barriers and facilitators to contraceptive implants). CR and CL met in person to discuss the results of the independent reviews, resolve a disagreement about study relevance in concert, and identify studies to move forward with. After the studies to be included in the final review were identified, CR tallied the specific mention of device-related and contraceptive medication−related barriers and facilitators in a word document. There was very little mention in the scientific literature about the device-related effects of contraceptive implants. Thus, stage 2 (summarized below) intended to learn more about users’ device-related subdermal implant experiences, as this will be important for our target population, GBM (stage 4).

### Stage 2: Review of YouTube Videos

A total of 2 members of the research team identified a collection of potentially relevant YouTube videos through a manual search. Search terms included lay and commercial names of contraceptives: “Nexplanon,” “Nexplanon insertion,” “Jadelle,” “Jadelle implant,” “Nexplanon birth control,” “Norplant,” “Implanon,” “Implanon birth control review,” and “Arm bar birth control.” We excluded sponsored testimonials, insertion or removal footage that included no assessment of the patient’s experience, clinical videos (ie, procedural videos on how to insert or remove implants), promotional or educational videos (ie, videos explaining what contraceptive implants are or how they work), and advertisements for any product. Then, spoken word transcripts of potentially relevant videos were automatically generated using YouTube’s state-of-the-art speech recognition algorithms [13].

To identify biomedical terms in YouTube transcripts, we used a publicly available natural language processing system called National Center for Biomedical Ontology Annotator, maintained by the National Library of Medicine [14]. The system recognizes sets of words and phrases using string matching and regular expressions that operate directly on text without preprocessing, such as spelling correction, part-of-speech tagging, and word sense disambiguation [15]. To identify the various side effects associated with subdermal implants, we utilized Systemized Nomenclature of Medicine (SNOMED) [16], a comprehensive terminology that provided term-to-concept mapping for a vast array of conditions, symptoms, and clinical findings.

We restricted our search to several types of concepts or *semantic types.* We identified relevant semantic types by using SNOMED to annotate a string of words typically associated with unpleasant experiences with drug delivery implants. We then reported the frequency of the specific terms describing these unpleasant experiences (eg, contusion and pain). Terms were paired to ensure clarity in the themes to which they were intended to refer (eg, bleeding, menstrual spotting). We then quantified the frequency (eg, number of videos) with which they appeared.

### Stage 3: In-Depth Interviews With Contraceptive Implant Users

In stage 3, we recruited potential participants using paid Facebook advertisements and posts on contraceptive implant-themed Facebook pages (eg, *Nexplanon* and *Implanon*) and other female-centered Facebook groups (eg, *Girls Love Travel*), with permission from page moderators. We exclusively sampled cisgender women in this stage because we wanted to ensure that the implant participants had used a design that was consistent with those of the PrEP implants in the development pipeline. Subdermal implants commonly available to men (eg, Testopel testosterone pellets, which are a nonremovable pellet implant, placed in the gluteal region) have a much different design [12,17]. This sampling approach had 2 purposes as it would allow (1) PrEP implant developers to incorporate feedback from past or current users of a similar implant into their device design and (2) the research team to be more precise about the questions asked to potential future PrEP implant users in the stage 4 web-based survey.

Participants were invited to click on a link that took them to a brief screening questionnaire to assess eligibility for telephone interviews about their experiences with implants. Eligible participants were at least 18 years old, identified as cisgender women, and had used an implant for contraception in the last 5 years. To sample a wide range of users’ experiences, we selected those who reported that they *liked very much* or *disliked very much* their implant on a 4-point Likert scale (1=disliked very much, 2=disliked a little, 3=liked a little, and 4=liked very much). In all, 24 participants were selected for interviews on a *first come first serve* basis, 12 who *liked very much* and 12 who *disliked very much* their implants and could be reached using the telephone numbers they provided.

Telephone interviews were audio-recorded and guided by findings from the YouTube analysis and scientific literature review. Specifically, we focused on the in-depth phone interview (IDI) guide on themes from the scientific literature and those YouTube users referenced frequently (identified using SNOMED). We categorized these themes into 6 broad categories: (1) general questions about participants’ experiences with subdermal implants, (2) decision making around the use of an implant, (3) acceptability of the implant, (4) the implantation process, (5) the removal process, and (6) recommendations for researchers working on a future PrEP implant. Participants were also asked about their reasons for removing the contraceptive implant. Upon completing the interviews, participants were compensated with US $50 Amazon gift cards.

Next, audio-recordings of the interviews were transcribed and cleaned for accuracy. To analyze the data, a codebook was developed jointly by the principal investigator of the project and a research assistant. It incorporated categories and themes from the interview guide and included definitions, inclusion and exclusion criteria, and examples of passages for inclusion. To validate and finalize the codebook, 2 researchers coded 2 transcripts independently, met to reconcile discrepant codes, and modified the codebook as needed to ensure that all themes of interest were being accurately captured. Researchers, then, proceeded to code the remainder of the transcripts in pairs, meeting after every 11 transcripts (half of the remaining data) were coded to reconcile discrepant codes. The finalized codes were analyzed using content analysis [18] to identify prevalent themes, illustrative examples of each theme, and knowledge gaps.

### Stage 4: Web-Based Surveys With Gay and Bisexual Men

Findings from the IDIs among cisgender women in stage 3 informed the development of a web-based quantitative assessment. This assessment took approximately 3 min to complete and aimed to determine how likely members of key HIV risk demographics (ie, GBM) would be to use an implant for HIV prevention. Specifically, we published text-based pop-up advertisements (known as broadcasts) on Grindr, a dating and *hookup* app for GBM. Logistically, these advertisements popped up the first time any Grindr user within a specified location opened the app; users who clicked on the advertisement were automatically rerouted to the quantitative assessment. For the purposes of this study, we published 2 broadcast advertisements (per city) in New York City, Baltimore, and Chicago. The quantitative assessment was open to anyone who wished to take it; eligible participants reported identifying as a gay man who was at least 18 years old. We focused our recruitment on GBM (rather than cisgender heterosexual cisgender women) because this population is at comparatively higher risk for HIV exposure and is one of the planned target populations for future PrEP implant studies. To reduce the odds of receiving multiple entries from the same person, participants did not receive compensation for their time, and surveys that had identical Internet Protocol addresses were eliminated. Additionally, for the purposes of the analyses presented in this paper, only HIV-negative GBM who lived in the United States were included.

As the PrEP implant is still hypothetical, we posed a variety of device configurations to participants. Specifically, the quantitative assessment measured how willing participants would be to use a hypothetical HIV prevention implant given a number of different factors: varying lengths of effectiveness (eg, 12 months, 6-8 months, and 2-3 months), number of implantable devices required, potential physical side effects (eg, bruising and scarring) resulting from the implantation or removal processes, implant size, and the convenience of using an implant compared with taking a daily HIV prevention pill (PrEP). All responses were recorded using a 5-point Likert scale, ranging from 1 (*I am very unlikely to use it)* to 5 (*I am very likely to use it)*. The value 3 on this scale represented a neutral response. Questions were presented in a different random order for each participant to minimize potential bias. Basic demographic, sexual risk, and injection drug use information were also collected using the web-based survey (Multimedia Appendix 1).

To give participants an idea of what a PrEP implant would entail, we embedded photos of the proposed implant next to a dime to help respondents estimate its size. Additionally, we embedded graphics interchange formats (GIFs: a lossless image file format that presents time-lapse video imagery) of the implant insertion and removal processes so that participants could gain an understanding of how they worked.

## Results

### Stage 1: Scientific Literature Review

In the scientific literature, all studies that were identified by the research team focused on cisgender women. Barriers to contraceptive implant uptake and sustained use most frequently included some that do not apply to an HIV prevention implant (eg, irregular or unpredictable menstrual bleeding [19-21]) and others that do apply, such as potential nonmenstrual side effects (eg, headache) [20,21]; perceived or actual high cost of implants [22-29]; patient or provider misconceptions or lack of information about implants (eg, believing that implants are contraindicated for specific patient populations) [21,23,26-32]; and challenges with access to implants [25,27]. Few studies discuss facilitators of the use of contraceptive implants as a birth control method and instead mostly focus on facilitators of implementation of programs designed to encourage implant use [26,33]. However, the scant body of existing evidence on implant facilitators shows that these include low implant cost, same-day insertion [27], and the inherent privacy of implants (eg, you can use this contraceptive method with low risk of others finding out) [29]. We were unable to identify scientific studies focused on user preferences regarding specific implant attributes (eg, size) or device-related effects (eg, bruising and local pain). Consequently, we sought additional sources of information.

### Stage 2: Self-Reported Side Effects Associated With Contraceptive Implants

Stage 2 summarizes our systematic review of YouTube videos. In July 2017, 272 videos about users’ experiences with contraceptive implants (that did not meet the exclusion criteria specified in the Methods section) had been uploaded to YouTube. Table 1 reports the frequency of videos in which the most often cited terms related to undesirable implant effects appear. These terms include the following: (1) pain, stinging, burning, or soreness and tenderness unrelated to menstruation (51 videos); (2) menstrual bleeding or spotting (33 videos); (3) contusion (32 videos); (4) scarring (25 videos); and (5) bleeding unrelated to menstruation (24 videos). We do not report demographic data as terms originated from videos posted on YouTube, and in the sample of videos accessed for the purposes of this study, posters rarely reported their demographic characteristics.

**Table 1:** Most frequently reported terms related to undesired implanted medication delivery device effects and the number of videos in which they are mentioned (stage 1; N=272 videos).

Term	Number of videos in which the term is referenced, n (%)
Pain, stinging, burning, or soreness and tenderness unrelated to menstrual bleeding or spotting	51 (18.8)
Menstrual bleeding or spotting	33 (12.1)
Contusion	32 (11.8)
Scarring	25 (9.2)
Bleeding unrelated to menstruation	24 (8.8)

### Stage 3: Users’ Reported Experiences With Contraceptive Implanted Medication Delivery Devices

Participants in IDIs were cisgender women who were aged, on average, 24 years and had used an implant for contraception in the last 5 years. Coders identified several themes within the topics previously recognized using SNOMED and covered in the IDI guide. Topics included the following: (1) specific attributes of implant products that attracted participants to these devices; (2) concerns about the implant; (3) feelings about the insertion process; (4) participants’ observed likes and dislikes about the implant, once it was inserted; and (5) recommendations for future implant designs and placement.

#### Specific Attributes of Implant Products That Attracted Participants

A majority of participants (n=20) reported that they were motivated to use contraceptive implants because they were long lasting and did not require additional attention or maintenance once inserted:

...I was on the pill for a long time and then I tried the shot and I didn’t like either of those options. So I kind of wanted something a little bit more permanent that I didn’t really have to worry about, which is why I thought it was a good fit for me...Like I said, it’s kind of more permanent, which I liked. You don’t really have to worry about it...

Participant 52: age 26 years, liked implant very much

I wanted something that I didn’t have to worry about...Like trying to remember to take a pill every day and, you know, this, you know, you don’t notice it’s there. You know, I mean, you can feel it if you touch it, but you don’t have to worry about anything. It’s just there...You don’t have to worry about taking a pill at the same time every day, or with the IUD, with it falling out, or something. I’ve heard stories of that. This you know is there. It’s not going to come out, unless you get it taken out.

Participant 46: age 36 years, liked implant very much

Almost half of the participants (n=10) reported that they chose implants because they believed that the side effect profile was favorable to other contraceptive medications on the market and even had some positive effects (eg, reduction in or stoppage of menstrual bleeding):

Well, it just seems less, like there’d be less side effects. Like, I have friends and stuff who’ve had the IUD and they say it causes really bad period cramps and I’ve never really heard of any bad stories with the Nexplanon, so that’s why I opted to go with it rather than the IUD.

Participant 96: age 25 years, liked implant very much

I liked the Nexplanon because there was a really high chance that I would not have a cycle, or I’d have a very light cycle because previous to that, I was having very heavy cycles, like painful, you know, just really couldn’t even go sit in class all day, I was cramping so much, stuff like that. So, I was hoping I’d be in that percentage of like, lightening up a little bit, or not having a cycle at all...

Participant 119: age 20 years, disliked implant very much

#### Concerns About the Subdermal Implant

Although many participants felt that the side effects of contraceptive implants were less problematic than those of other contraceptives, they still expressed some concerns (n=12):

And then some of the cons that I had heard were weight gain, depression, mood swings, stuff like that. But at the time -- you hear different things about different birth controls and everybody’s body’s different so even though I had heard those few bad things I was still willing to give it a try.

Participant 37: age 20 years, disliked implant very much

…I anticipated highly irregular periods. I also anticipated looking for any sort of hormonal imbalances or emotional imbalances based on hormone usage. Nexplanon is my first birth control, so since I didn’t have any prior experience I was just wary of hormonal medication at all...

Participant 138: age 23 years, liked implant very much

However, given that PrEP implants do not contain hormonal medications, these concerns would not apply.

#### Feelings About the Insertion Process

Many participants (n=15) felt that the insertion process was quick and straightforward:

it’s very straightforward. It’s really like a two-process insertion, which I thought was great, rather than them trying to feel around where to put it. They insert the little machine and click it in and that’s it. So it’s very simple...

Participant 56: age 26 years, liked implant very much

...I mean, it was pretty easy for them to...get it in there with the -- I guess they use a needle after they numb you, and they make the incision -- I guess it’s a needle, I don’t really -- it’s a big one, but it’s pretty easy to put in there...I guess when they made that incision, and then used the little insert - that was pretty fast. Like, it -- when they put it in it goes really fast. It’s just waiting to be numb that took the longest.

Participant 260: age 20 years, disliked implant very much

Participants reported few dislikes about the insertion process, though a minority (n=5) felt intimidated by the size of the implant or applicator:

Yeah, like I mentioned, whenever you first see it -- and I’m not a big needle person, anyways, but whenever you first see it, it looks scary. The needle that they insert it with is pretty wide.

Participant 37: age 20 years, disliked implant very much

After the device was inserted, the majority of participants (n=21) experienced localized pain related to device insertion. This pain was mostly mild and included bruising, soreness, and bleeding:

Immediately, the area was like kind of a little bit sore, but like I said, it just like bruised a little bit and then it was fine...It was kind of bruised like for a couple of days, but after that it was fine.

Participant 52: age 26 years, liked implant very much

My arm bled for a little while after they got it in, so I had to keep the wrap on for three days and it was a really smooth transition putting it in...They recommended to keep it on for three days, but it was really, really tight, so it was a little rough to keep the wrap on for three days, and showering and moving around with a tight, brown wrap on your arm. So I did end up taking it off, I think, after two. But by then, it was fine.

Participant 16: age 24 years, disliked implant very much

#### Participants’ Likes and Dislikes About the Implant After It Was Inserted

After the implant was inserted, participants continued to view the long-lasting properties of the device and its little need for user attention as a benefit (n=13):

I liked that I didn’t have to take something every day, that it was just there...but pretty much I didn’t have to remember to take anything and I knew I wasn’t going to get pregnant because it had worked for so many other girls before.

Participant 64: age 27 years, liked implant very much

...Overall, I feel it’s been more beneficial to my life than the pill was. Because, once again, convenience. I don’t have to forget anything. I’ve got the little card in my wallet that says, “OK, this is when you need to get it removed.” It’s in my arm, it’s there, good to go...

Participant 98: age 19 years, liked implant very much

Additionally, almost half of the participants (n=10) liked the implant’s effectiveness in preventing pregnancy:

...I like the fact that I know that I could have unprotected sex with this, and I won’t get pregnant. And I don’t have to use condoms, because it’s 100 percent active, like effective...I know I’m not going to get pregnant because I don’t need that, especially after I just had a baby not too long ago...

Participant 235: age 30 years, disliked implant very much

Participants liked the implant’s small size (n=13):

It’s small...So, if it would have been something a bigger size, I probably wouldn’t have put it on, because I -- to have this thing stay in my arm, it would have been probably maybe uncomfortable, but the fact that it’s small, it -- I can’t feel it. It’s not heavy.

Participant 86: age 28 years, liked implant very much

...it was smaller. I wouldn’t have to worry about anybody really noticing, or being able to associate anything with having birth control... It just made me feel more comfortable that it was smaller, and that I wouldn’t have to worry about if it was large or thick, restraining any activity, or anything like that, that it might bend or break or anything.

Participant 201: age 20 years, disliked implant very much

Furthermore, participants liked that they could not feel the device while they carried out their normal daily activities (n=14):

I think, also the fact that you could tell like it was flexible, my job that I had, I would have to pull out of boxes that were kind of almost about like chest height, and I – there was one time I bent in, and I felt it hit against my arm, but because of it being flexible, it just kind of went with it instead of it had been more sturdy or like, more structured, I felt like it could have possibly broken in that situation just because I had forgotten about it and didn’t even think about reaching in like that.

Participant 68: age 24 years, disliked implant very much

Although participants liked the small size of the device and that it was relatively unnoticeable during normal activities, some participants (n=8) also liked that the device was detectable if they searched for it. They found this reassuring, as they could ensure that the device was still in the correct spot and had not migrated:

You can’t -- with an IUD, you can’t check to see if it has moved, really. I mean, you kind of can, but not really. With this implant, you can feel where it is. If it feels like it’s starting to, like, imbed further, I could go into the OB and have them check to make sure it hasn’t moved.

Participant 67: age 23 years, liked implant very much

...*it doesn’t bother me. I can poke it, and I can see it, but other than that it doesn’t bother me. It’s just there...It doesn’t weird me out or anything. It’s normal. I got used to it. It was kind of weird at first. But as long as I know it’s there, and it’s not like going further into my tissue, then it makes me a lot more comfortable knowing it’s actually still there. Because I’ve heard the horror stories of having to get it taken out and having to have an actual surgery to get it out versus it just being pulled out.* [Participant 211: age 24 years, disliked implant very much]

Some participants (n=8) reported that the implant felt uncomfortable when bumped:

...If I bump into anything on that arm where the implant is, it’s uncomfortable, but it doesn’t swell or anything like that...When I bump it it’s just kind of a dull pain, like almost like a bruise feels. It’s kind of weird to explain because it’s internal pain, but it’s -- I don’t know, it’s kind of weird. It’s like when you bruise your foot and you keep bumping it, it feels like that for a short while.

Participant 98: age 19 years, liked implant very much

It’s a thin rod. If it was any bigger, I think they would probably bother people because if I put pressure on it -- you know, like, sometimes you turn around or something in the car -- not while you’re driving, obviously, but I’ll turn around in the passenger seat to check on my kids. And if you push your arm where the implant is up against something, it will start to hurt. It’ll start to irritate me, so I try not to put pressure on the implant.

Participant 67: age 23 years, liked implant very much

#### Recommendations for Future PrEP Implant Designs and Placement

Participants had suggestions for researchers regarding the design of the PrEP implant device. Specifically, some participants wanted the device to be designed in a way that would facilitate easier removal (n=7), be small in size, constructed from a flexible material (n=15), and be placed in an area of the arm that would not interfere with the device (n=9):

...*Maybe make it easier to remove if there’s any way to do that. I think that’s the only thing that’s really deterring people is that they’re scared it’s going to get stuck...I’ve seen pictures where they’ve cut people a couple of times, and they can’t seem to get it out. So if there’s any way to make sure that it’s going to be easy to come out with this design you guys are making -- I don’t know if there’s a way to do that, but...I wish they could remove it like that with the needle instead of cutting your arm.* [Participant 67: age 23 years, liked implant very much]

It’s like a slim rod that’s really lightweight and plastic so like you don’t feel it, and it just comes -- so, it goes in easily, but yeah, if it was shorter, it probably would have been a little more appealing...I don’t really have an opinion about the size. I mean, it was I think a couple inches long, which is kind of big, and I probably would have liked it to be a little smaller, but it still wasn’t too bad.

Participant 66: age 21 years, liked implant very much

I don’t feel like it hit on anything; I don’t feel like it would hit against like the underwire of my bra because of where it was placed. And so, it was very convenient because it was just out of the way...I’d say keep the flexibleness of that, but just kind of, make sure it’s like as in-invasive to somebody’s day-to-day life as possible. So, placed in a spot that isn’t going to affect people that have different lifestyles, or maybe even have different options based on people’s lifestyles and what they do, that that could be a possibility too.

Participant 68: age 24 years, disliked implant very much

### Stage 4: Gay and Bisexual Men’s Likelihood of Using a PrEP Implant

Participants (N=304) in the stage 4 web-based survey were aged, on average, 38 years and predominantly self-identified as gay or homosexual (204/238, 85.7%) cisgender men (231/238, 97.1%); about half (125/238, 52.5%) of them identified as white and a quarter (58/238, 24.4%) identified as Latino. Over two-thirds of the sample were college graduates (89/238, 37.4%) or had attended graduate school (77/238, 32.4%) and reported ever (101/174, 43.4%) or currently (73/174, 30.7%) using oral PrEP. Condomless sex with an HIV-positive or unknown status partner (104/126, 43.7%) was the most frequently reported HIV risk behavior in the past year (Table 2).

**Table 2:** Demographic information (stage 4; N=238).

Sample characteristics		Value, mean (SD)	Value, range	Value, n (valid %)^a^
Age (years; N=238)		36.44 (11.33)	18-71	N/A^b^
**Race/ethnicity (N=238)**				
	White	N/A	N/A	125 (52.5)
	Latino	N/A	N/A	58 (24.4)
	African American	N/A	N/A	27 (11.3)
	Asian or Pacific Islander	N/A	N/A	19 (8.0)
	Other	N/A	N/A	9 (3.7)
**Education (N=238)**				
	College graduate	N/A	N/A	89 (37.4)
	Graduate school	N/A	N/A	77 (32.4)
	Some college	N/A	N/A	40 (16.8)
	Trade/Technical/Vocational	N/A	N/A	12 (5.0)
	High school graduate/general education diploma	N/A	N/A	19 (8.0)
	Less than high school	N/A	N/A	1 (0.4)
**Gender identity (N=238)**				
	Man	N/A	N/A	231 (97.1)
	Transgender, gender queer, nonbinary	N/A	N/A	7 (3.0)
**Sexual orientation (N=237)**				
	Gay or homosexual	N/A	N/A	204 (86.1)
	Bisexual	N/A	N/A	28 (11.8)
	Straight or heterosexual	N/A	N/A	2 (0.8)
	Other	N/A	N/A	3 (1.3)
**Pre-exposure prophylaxis** **(N=174)**				
	Ever used	N/A	N/A	101 (58.0)
	Currently using	N/A	N/A	73 (42.0)
**HIV risk behaviors in past year (N=126)**				
	Condomless sex with HIV-positive or unknown status partner	N/A	N/A	104 (82.5)
	Traded sex for money or other goods or services	N/A	N/A	16 (12.7)
	Injected drugs	N/A	N/A	6 (4.8)

^a^Valid percent is the percent when missing data are excluded from the calculation.

^b^N/A: not applicable.

Table 3 shows the participants’ preferences related to specific PrEP implant characteristics. Of these characteristics, not having to take a daily pill increased the likelihood of PrEP implant use (mean 4.13). Requiring more than 1 device to achieve 1 year of protection (mean participant rating range:1.79-2.94) mildly discouraged implant use. Participants did not mind the following: moderate bruising, a small scar, tenderness, or bleeding after insertion or removal, or having an implant slightly longer or fatter than a matchstick.

**Table 3:** Participants’ likelihood of using pre-exposure prophylaxis implants based on their specific potential attributes (stage 4; N=304).

Implant characteristic		Likelihood of use^a^	
**Duration of protection, mean (SD)**			
	Protection lasts up to 12 months		3.6 (1.5)
	Protection lasts up to 6-8 months		3.3 (1.5)
	Protection lasts up to 2-3 months		2.7 (1.6)
**Number of implants needed, mean (SD)**			
	2 implants needed		2.9 (1.6)
	3 implants needed		2.2 (1.4)
	4 implants needed		1.8 (1.3)
**Potential application site related−side effects, mean (SD)**			
	Moderate bruising		3.7 (1.4)
	Small scar		3.5 (1.5)
	Implant can be felt		3.5 (1.5)
	Some pain		3.6 (1.4)
	Bleeding		3.6 (1.4)
	Tenderness		3.5 (1.4)
**Other PrEP^b^ implant device–related characteristics, mean (SD)**			
	No daily oral PrEP		4.1 (1.3)
	Trocar is used to insert implant		3.7 (1.4)
	Must go to the doctor’s office for removal		3.9 (1.4)
	Implant longer than a matchstick		3.5 (1.4)
	Implant fatter than a matchstick		3.2 (1.4)

^a^Values presented are descriptive statistics only; value of 3 on the Likert scale indicates a neutral rating.

^b^PrEP: pre-exposure prophylaxis.

## Discussion

The scientific literature review conducted in stage 1 did not yield data on specific implant *device-related* barriers or facilitators. However, the existing literature did show that many of the problems associated with *general* contraceptive implant use are similar to those observed in relation to oral PrEP use. For example, several studies identified patient or provider misconceptions or lack of information about implants as a barrier to their uptake. Specifically, patients who have providers who harbor misconceptions about PrEP (eg, PrEP is only moderately effective) are less likely to obtain a prescription for this medication from their provider [34]. Additionally, concerns about contraceptive implants’ side effect profiles and potential high cost deterred use; these same barriers also affected oral PrEP use [35]. Finally, access to contraceptive implants or oral PrEP was a challenge to use for each of these products [36]. All these concerns are likely to translate to PrEP implants. Suggestions from the contraceptive and oral PrEP studies on how to overcome these issues may be helpful. For instance, concerted efforts (eg, trainings) to ensure that providers have accurate information about PrEP [37,38] implants (including information on insertion and removal techniques) and effective tools to identify PrEP implant candidates could be helpful. Same-day access to PrEP implant insertion could also boost uptake [39]; offering PrEP implants in emergency department settings could also boost implant uptake.

Potential device side effects were a concern, as expressed by participants in stages 2, 3, and 4. Specifically, in stage 2, YouTube videos identified a number of potential side effects related to contraceptive implant use that could potentially be concerns for PrEP implant users, including pain, stinging, burning, soreness or tenderness, contusion, scarring, and bleeding. Participants in stage 3 also reported these effects after contraceptive implants were inserted or removed, although this was typically mild. Results from stage 4 showed that the possibility of experiencing these effects (eg, moderate bruising, small scar, some pain, bleeding, and tenderness) did not impact GBM participants’ likelihood of using PrEP implants. Although these outcomes may not present a barrier to use for these men, this is not the case for all potential users. For example, other studies have shown that some transgender women were concerned that mild scarring and other skin-related reactions related to potential future PrEP implants could become serious [40,41]. Thus, it may be necessary to engage novel strategies to communicate actual risk [41], as some populations may overestimate this. Icon arrays, which communicate risk using graphical representations of icons symbolizing people affected by a given outcome (eg, circles), may be one such strategy [42]. Icon arrays are shown to be better at communicating risk for low-risk procedures and may be especially effective for populations that have variability in numeracy skills [43].

Stage 2 YouTube videos reported menstrual complaints related to contraceptive implants. Stage 3 interviews also revealed that contraceptive implants had a negative effect on menstruation for some users. Specifically, over half of the participants endorsed this idea, complaining of prolonged or unpredictable cycles. It is highly unlikely that the menstrual or hormonal issues identified in stages 2 and 3 would be problematic for PrEP implant users, as these effects are associated with the use of hormonal medications, not antiretrovirals. However, given that this is such a pervasive issue among contraceptive implant users, it is paramount to ensure that potential PrEP implant users do not attribute these effects to implants in general. Rather, we must clarify that implants are a method to distribute a variety of medications. They are not a medication themselves. Potential PrEP implant users must be made aware that hormonal medications used in contraceptive implants, and antiretroviral medications used in PrEP implants will have completely different effects from one another. Communicating this will be particularly important for cisgender and transgender women users, as they may have greater familiarity with contraceptive implants and may be more concerned about how PrEP implants would interact with female hormones. Stage 4 participants were not asked about this theme because (1) this concern would not apply to male users and (2) medications contained in PrEP implants are not anticipated to interfere with male hormones.

Although negative side effects can deter implant use, other side effects may be seen as positive. Nearly half of the participants in stage 3 revealed that they chose the contraceptive implant because they believed that it had a more favorable side effect profile than other available options. Although specific side effects of the antiretroviral drug that would be delivered via implant are still under study (eg, these side effects may be different, and data show that these drugs are well tolerated [44,45]), it is critical to consider their side effects parallel to the HIV prevention effects of these medications. Users’ experiences with oral PrEP show that this is the case; fear or experience with unfavorable short- (eg, gastrointestinal effects) or long-term side effects (eg, renal toxicity) are factors for drug discontinuation in 9.2%-33.0% of GBM who stop taking PrEP [46-48]. implants that distribute PrEP medications that have side effect profiles perceived as too intolerable may have limited uptake and continued use.

Conversely, if they seem to have fewer or beneficial side effects due to the delivery method, this could be viewed as positive. For instance, some stage 3 participants reported using contraceptive implants because they have the potential to have the beneficial side effect of lessened menstruation. Although it is highly unlikely that PrEP implants would have a similar effect, it is important to note the appeal of beneficial side effects. This is particularly important for those beneficial side effects that produce a notable enhancement in the life or appearance of the user, since these enhancements could potentially facilitate improved uptake or sustained use. Beneficial side effects have been noted in other medications, including finasteride (hair regrowth in men experiencing male pattern baldness [49] and initially used to treat benign prostatic hyperplasia [50]) or oral contraceptives (facial acne reduction and initially used to prevent pregnancy [51]). If and when beneficial side effects are identified in drugs contained in PrEP implants, this could play a role in future messaging and uptake campaigns.

The effectiveness and duration of products as well as the amount of maintenance they would require were important to participants in stages 2-4. Specifically, participants in stage 3 liked that the contraceptive implant was effective at preventing pregnancy and lasted for a prolonged period, during which little maintenance was required on their behalf (eg, they did not have to perform a routine activity, such as taking a daily pill). Participants in stage 4 shared this sentiment, and not having to take a daily pill increased the likelihood of using the PrEP implant. Additionally, in stage 4, protection against HIV lasting 6 months or more modestly increased potential users’ likelihood of implant use; protection lasting 2-3 months modestly decreased the likelihood of use. Given this, it is critical in development phases to prioritize the amount of time that the implant will last and the amount of maintenance it requires.

The size and number of devices were also important. For example, in stage 3, participants found the small size and unintrusiveness (eg, most participants could not feel the implant during daily activities) of the contraceptive implant to be positive attributes. In stage 4, participants found the potential implant size (slightly longer and fatter than a matchstick) neither motivated nor dissuaded the likelihood of use. This may mean that the current size is acceptable. However, reducing the size of the implant could have a positive impact on potential users’ perceptions of it. Related to this, stage 4 participants also reported that as the number of implants necessary to provide protection against HIV increases, the likelihood of use of this prevention strategy decreases. Taken together, this means that implant size and the number of implants necessary to achieve HIV protection are also critical factors for potential user uptake. The trajectory of contraceptive implant development shows that although it may not be possible to devise a small, 1-device implant in the first product iteration, it is possible to reduce implant size and number over time [11,52]. Thus, developers of PrEP implants should aim to reduce the size and number of devices, even if this is not immediately possible.

Participants in stage 3 (all cisgender women) reported that the insertion and removal procedures for contraceptive implants were quick and straightforward, with minimal pain. There was no mention of discomfort related to having this procedure done in a health care provider’s office. However, a minority of participants reported feeling intimidated by the size of the trocar used to insert the contraceptive implant. This was not the case in stage 4. Stage 4 participants (all GBM) did not find the potential trocar planned for use in clinical research to implant the PrEP implant to be a barrier to uptake. It is important to note that no participants in stage 4 had ever undergone this procedure, although stage 3 participants had. This could have contributed to differences in participants’ level of comfort with trocar insertion.

Furthermore, having the implant t removed by a health care provider appeared to mildly improve the likelihood of use among stage 4 participants. Thus, it appears that users of contraceptive implants and potential users of PrEP implants are mostly comfortable with the proposed model for device application and removal. However, although we can only speculate, it may be that this level of comfort is based on the proposed length of time that the implant could be used. That is, potential users may be less likely to undergo these insertion and removal processes if the implant does not last for very long. This again highlights the importance of the device duration.

### Limitations

In stage 2, we may have missed some references to specific participant experiences if participants used descriptive words not contained in SNOMED’s database. The interviews we conducted as part of stage 3 were partially meant to address this potential gap by more fully fleshing out users’ experiences with contraceptive implants. As is the case with all research on acceptability and feasibility of hypothetical clinical products, it is difficult to estimate the gap between participants’ perceived likelihood of use and actual use of implants for PrEP. However, data from past and current users of similar implants for contraception in stages 2 and 3 help to mediate this issue.

Additionally, because we were concerned with ensuring that users’ experiences with existing implants were as similar as possible to potential future PrEP implants, we sampled only women in stage 3. In stage 4, we targeted only GBM (as they have a higher risk for HIV exposure compared with heterosexual cisgender women) with web-based surveys on their preferences for PrEP implants. Given the lack of formative work specific to GBM, there may be a gap in the preferences and perceptions of this implant product between these 2 groups. Future studies should conduct qualitative interviews with GBM on their preferences around PrEP implant products to limit this shortcoming. Despite these limitations, given the dearth of research on this topic, this work makes an important and unique contribution to the literature on implants for PrEP.

### Conclusions

In all stages of research, participants reported experiencing (stages 2 and 3) or anticipating (stage 4) implant-related side effects. These effects were related to the device itself (bleeding, bruising, and pain after insertion) or, among contraceptive implant users, medication-related side effects (menstrual changes, weight gain, and acne). Although it is highly unlikely that PrEP implant users will experience similar hormonal side effects to contraceptive implant users, it is critical to point out the difference between these 2 products to potential PrEP users. Icon arrays could be a useful way to communicate serious risks related to implant devices. Future studies should monitor for the presence of beneficial side effects related to implant medication, as this has been evidenced with other medications and may improve uptake and sustained use. Furthermore, small implants need only 1 device, last for at least six months, are unintrusive, and require little to no user effort to optimize uptake. Current and potential future users appear comfortable with the currently used procedures for implant insertion and removal. Finally, future work should include more formative work specific to GBM, as this will allow researchers to address their concerns with greater precision.

## Appendix

Multimedia Appendix 1 Web-based survey.

## Abbreviations

GBM: gay and bisexual men
IUD: intrauterine device
IDI: in-depth phone interview
PrEP: pre-exposure prophylaxis
SLAP-HIV: Sustained Long-Action Prevention Against HIV
SNOMED: Systemized Nomenclature of Medicine
