# Supplementary material for: Understanding the Acceptability of Subdermal Implants as a Possible New HIV Prevention Method: Multi-Stage Mixed Methods Study
Source: J Med Internet Res. 2020 Jul 27;22(7):e16904. doi: 10.2196/16904 (PMC7418007; doi:10.2196/16904)
Supplement: Multimedia Appendix 1 [file jmir_v22i7e16904_app1.pdf]

HIV Implant User's Experience Survey

SCREENING

This research study is led by Columbia University scientists. By clicking NEXT, you acknowledge that you are 18 years old or older: *[next]*

INTRODUCING THE IMPLANT

Screen 1: INTRODUCTION: Thank you for participating in this confidential survey. It takes less than 10 minutes. First, we will present information on an implant in development to prevent HIV. Next, you will be asked for your opinions.

Screen 2: WHAT IS THE IMPLANT?
The implant….

- Is similar to other implants already used for birth control
- Is a device that slowly releases medicine to protect against HIV infection for up to 12 months
- Is slightly larger than a matchstick
- Is placed under the skin on the inside of your upper arm
- Must be removed and a new one inserted after 12 months

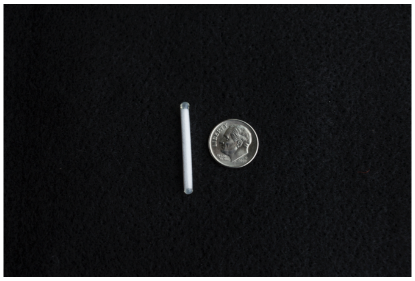

Screen 3: HOW DOES IMPLANT INSERTION AND REMOVAL WORK?

- To put the implant in, a healthcare provider first applies pain numbing medicine so you don't feel anything; then, they use a special applicator to slide the implant under your skin
- To take the implant out, a healthcare provider makes a small incision (no more than ¼ inch) and the implant is removed with tweezers
- A new implant can be put in through the same incision. Or, a new implant could be put in the other arm through a new incision

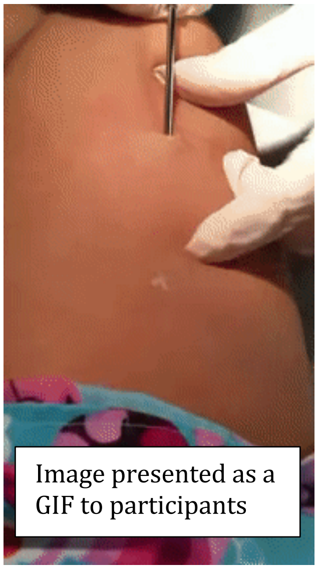

Image presented as a GIF to participants

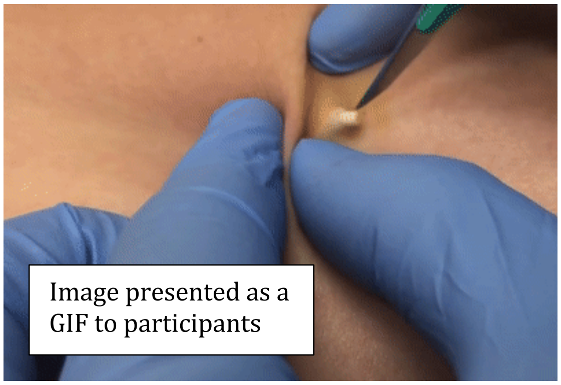

Image presented as a GIF to participants

Screen 4: CAN YOU SEE OR FEEL THE IMPLANT?

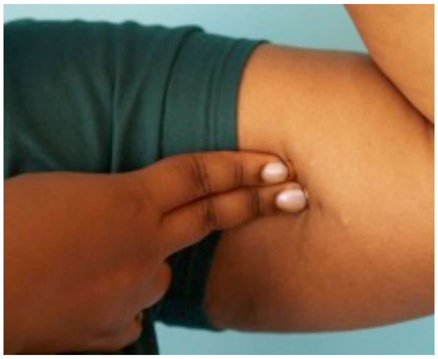

Most people who have used similar implants (e.g., for birth control) say…

- You cannot feel the implant unless you press down on the skin above it
- The implant is not painful. In fact, most people say the device is flexible and comfortable
- The implant is not very noticeable. It may be possible to see it by looking closely at the area of the arm where it is inserted

Screen 5: ARE THERE SIDE EFFECTS FROM THE INSERTION OR REMOVAL PROCEDURE?
Side effects are possible. However, most side effects are rare and mild. They might include the following at the insertion/removal site…

- Mild to moderate pain
- Some bleeding
- A small scar (usually about ¼ inch)
- Infection

Screen 6: HOW MUCH DOES THE IMPLANT COST?

- Cost will depend on insurance coverage, and in some cases, the implant may be covered by the government or drug manufacturer

Screen 7: CONCLUSION: Thanks for learning about this implant. Now, we will ask your opinion on some of the implant's characteristics and potential side effects. We will also ask you about yourself to identify ways to make the implant better for different groups of people. We will not reveal your identity.

SURVEY

Using the scales below, please indicate how much each potential implant characteristic factors into your likelihood of using it.

1. Overall, how likely would you be to use an implant that would be placed under the skin of your arm and would protect you against HIV …

   a. for ***up to 12 months***?

1-------------------------2-------------------------3-------------------------4-------------------------5

I am very unlikely to use it	I am somewhat unlikely to use it	I am neither likely nor unlikely to use it	I am somewhat likely to use it	I am very likely to use it

   b. for ***up to 6-8 months***?

1-------------------------2-------------------------3-------------------------4-------------------------5

I am very unlikely to use it	I am somewhat unlikely to use it	I am neither likely nor unlikely to use it	I am somewhat likely to use it	I am very likely to use it

   c. … for ***up to 2-3 months***?

1-------------------------2-------------------------3-------------------------4-------------------------5

I am very unlikely to use it	I am somewhat unlikely to use it	I am neither likely nor unlikely to use it	I am somewhat likely to use it	I am very likely to use it

2. Scientists anticipate that you will need ***one implant*** to protect you against HIV for 12 months. However…

   a. …what if you need ***two implants*** at one time?

1-------------------------2-------------------------3-------------------------4-------------------------5

I am very unlikely to use it	I am somewhat unlikely to use it	I am neither likely nor unlikely to use it	I am somewhat likely to use it	I am very likely to use it

   b. …what if you need ***three implants*** at one time?

1-------------------------2-------------------------3-------------------------4-------------------------5

I am very unlikely to use it	I am somewhat unlikely to use it	I am neither likely nor unlikely to use it	I am somewhat likely to use it	I am very likely to use it

   c. …what if you need ***four implants*** at one time?

1-------------------------2-------------------------3-------------------------4-------------------------5

I am very unlikely to use it	I am somewhat unlikely to use it	I am neither likely nor unlikely to use it	I am somewhat likely to use it	I am very likely to use it

3. After having a similar implant *inserted* for contraception…

a. some people report mild to moderate bruising lasting 7 days or less. How would this factor into your likelihood of using an implant for HIV prevention?

1-------------------------2--------------------------3-------------------------4--------------------------5

I am very unlikely to use it	I am somewhat unlikely to use it	I am neither likely nor unlikely to use it	I am somewhat likely to use it	I am very likely to use it

b. some people report that they have a small scar on the inside of their arm that is less than ¼ inch long. How would this factor into your likelihood of using an implant for HIV prevention?

1-------------------------2--------------------------3-------------------------4--------------------------5

I am very unlikely to use it	I am somewhat unlikely to use it	I am neither likely nor unlikely to use it	I am somewhat likely to use it	I am very likely to use it

c. some people report that they can feel the implant when they press down on the skin on top of it and that this is not painful or bothersome. How would this factor into your likelihood of using an implant for HIV prevention?

1-------------------------2--------------------------3-------------------------4--------------------------5

I am very unlikely to use it	I am somewhat unlikely to use it	I am neither likely nor unlikely to use it	I am somewhat likely to use it	I am very likely to use it

d. some people report mild pain or pressure during the insertion and removal processes. How would this factor into your likelihood of using an implant for HIV prevention?

1-------------------------2--------------------------3-------------------------4--------------------------5

I am very unlikely to use it	I am somewhat unlikely to use it	I am neither likely nor unlikely to use it	I am somewhat likely to use it	I am very likely to use it

e. some people report mild bleeding during the insertion and removal processes. How would this factor into your likelihood of using an implant for HIV prevention?

1-------------------------2--------------------------3-------------------------4--------------------------5

I am very unlikely to use it	I am somewhat unlikely to use it	I am neither likely nor unlikely to use it	I am somewhat likely to use it	I am very likely to use it

f. Some people report pain or tenderness lasting 2-3 days after insertion and removal. How would this factor into your likelihood of using an implant for HIV prevention?

1-------------------------2--------------------------3-------------------------4--------------------------5

I am very unlikely to use it	I am somewhat unlikely to use it	I am neither likely nor unlikely	I am somewhat likely to use it	I am very likely to use it

4. People who use the HIV prevention implant will not have to remember to take an HIV prevention pill (also known as the PrEP pill) on a daily basis. How would this factor into your likelihood of using the implant?

1-------------------------2--------------------------3-------------------------4--------------------------5

I am very	I am somewhat	I am neither	I am somewhat	I am very likely

unlikely to use it unlikely to use it likely nor unlikely to use it likely to use it to use it

5. The tool used to insert the implant could look like this:

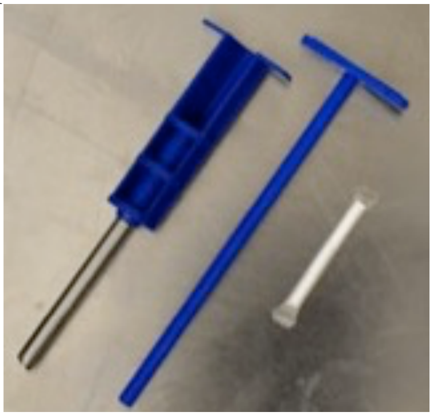

How would this factor into your likelihood of using it?

1-------------------------2--------------------------3-------------------------4--------------------------5

1	2	3	4	5
I am very unlikely to use it	I am somewhat unlikely to use it	I am neither likely nor unlikely to use it	I am somewhat likely to use it	I am very likely to use it

6. The implant must be inserted and removed in a healthcare provider's office. How would this factor into your likelihood of using it?

1-------------------------2--------------------------3-------------------------4--------------------------5

1	2	3	4	5
I am very unlikely to use it	I am somewhat unlikely to use it	I am neither likely nor unlikely to use it	I am somewhat likely to use it	I am very likely to use it

to use it

7. The implant may be slightly longer than a matchstick. How would this factor into your likelihood of using it?

1-------------------------2--------------------------3-------------------------4--------------------------5

1	2	3	4	5
I am very unlikely to use it	I am somewhat unlikely to use it	I am neither likely nor unlikely to use it	I am somewhat likely to use it	I am very likely to use it

   a. What if the implant were slightly fatter than a matchstick. How would this factor into your likelihood of using it?

1-------------------------2--------------------------3-------------------------4--------------------------5

1	2	3	4	5
I am very unlikely to use it	I am somewhat unlikely to use it	I am neither likely nor unlikely to use it	I am somewhat likely to use it	I am very likely to use it

Demographic questions

Please answer the following questions about yourself. Remember that study staff do not have personally identifying information about you.

8. How old are you?

a. _ _ [range: 00-99]

9. What is your race/ethnicity? (Please choose one.)
   a. African American/Black
   b. Latino
   c. White
   d. Asian/Pacific Islander
   e. Native American
   f. Other

10. What is the highest level of education you have completed?
    a. Less than high school
    b. High school graduate/GED
    c. Some college
    d. Trade/technical/vocational school
    e. College graduate (Bachelor's degree)
    f. Graduate/professional school

11. What sex were you assigned at birth on your original birth certificate?
    a. Male
    b. Female

12. Do you currently identify as a man, a woman, transgender, or another gender?
    a. Man
    b. Woman
    c. Transgender
    d. Genderqueer/non-binary
    e. Another gender

13. What best describes your current sexual orientation?
    a. Gay, Lesbian, Queer, Homosexual
    b. Bisexual
    c. Straight, Heterosexual
    d. Another orientation

14. Have you ever tested HIV positive?
    a. Yes
    b. No
    c. Refuse to answer

15. Have you ever used PrEP?
    a. Yes [SKIP TO Q16]
    b. No

16. Are you currently using PrEP?
    a. Yes
    b. No

17. In the last year, have you had sexual intercourse (anal or vaginal) without a condom with somebody who is HIV positive or whose HIV status you did not know (meaning they could not present you with HIV test results that were less than 3 months old)?
    a. Yes
    b. No

18. When you had sexual intercourse (anal or vaginal) in the last year how frequently do you use condoms?
    a. Never
    b. Rarely
    c. Most of the time
    d. Always
    e. I have not had sex in the last year

19. Considering your usual sexual behavior, how likely is it that you will get HIV in your lifetime?

1----------2----------3----------4----------5----------6----------7----------8----------9----------10

Extremely unlikely ... Extremely likely

20. Considering your usual sexual behavior, how likely is it that you will get a sexually transmitted disease (STD), not including HIV in the next year?

1----------2----------3----------4----------5----------6----------7----------8----------9----------10

Extremely unlikely ... Extremely likely

21. In the last year, have you injected drugs?
    a. Yes
    b. No

22. In the last year, have you traded sexual activity or favors for food, money, a place to sleep, drugs, or other material goods?
    a. Yes
    b. No

23. Do you live in the United States?
    a. Yes
    b. No **[SKIP to Q25]**

24. What is your current 5-digit zip code _________ **[SKIP to end of survey]**

25. What geographic region do you currently live in? [Dropdown menu]
    a. Africa
    b. Asia
    c. Central America
    d. Eastern Europe
    e. European Union
    f. Middle East
    g. North America
    h. Oceania
    i. South America
    j. Caribbean

Thank you! We appreciate you taking the time to complete this survey. Your answers are important to us.

Click here if you wish to be contacted for other studies: *[checkbox]*

FOR PARTICIPANTS WHO WISH TO BE CONTACTED FOR OTHER STUDIES:
Thank you! Please leave your contact information below for us to follow up with you.

Name: (required)
City: (required)

State: (required)
Email address: (required)
Telephone number: (required)
